# Production and performance evaluation of biodiesel from *Elaeis guineensis* using natural snail shell-based heterogeneous catalyst: kinetics, modeling and optimisation by artificial neural network

**DOI:** 10.1039/d3ra02456c

**Published:** 2023-06-28

**Authors:** Chinwe P. Okonkwo, Vincent I. E. Ajiwe, Alexander I. Ikeuba, Wilfred Emori, Modestus O. Okwu, Jude I. Ayogu

**Affiliations:** a Department of Pure and Industrial Chemistry, Nnamdi Azikiwe University Awka Nigeria cp.okonkwo@unizik.edu.ng; b Materials Chemistry Research Group, Department of Pure and Applied Chemistry, University of Calabar Calabar Nigeria; c School of Materials Science and Engineering Sichuan University of Science and Engineering Sichuan Province PR China; d Department of Mechanical Engineering, Federal University of Petroleum Resources Effurun Ugbomoro Nigeria; e Department of Pure and Industrial Chemistry, University of Nigeria Nsukka 410001 Nigeria jude.ayogu@unn.edu.ng; f Department of Chemistry, Faculty of Mathematical and Physical Science, University College London London WC1E 6BT UK

## Abstract

This study presents an approach to produce biodiesel from *Elaeis guineensis* using natural heterogeneous catalysts derived from raw, calcined, and acid-activated forms of waste snail shells. The catalysts were thoroughly characterized using SEM, and process parameters were systematically evaluated during biodiesel production. Our results demonstrate a remarkable crop oil yield of 58.87%, with kinetic studies confirming second-order kinetics and activation energies of 43.70 kJ mol^−1^ and 45.70 kJ mol^−1^ for methylation and ethylation, respectively. SEM analysis identified the calcined catalyst as the most effective, exhibiting remarkable reusability for continuous reactions running up to five times. Moreover, the acid concentration from exhaust fumes yielded a low acid value (B100 0.0012 g dm^−3^), significantly lower than that of petroleum diesel, while the fuel properties and blends satisfied the ASTM standards. The sample-heavy metals were well within acceptable limits, confirming the quality and safety of the final product. Our modelling and optimization approach produced a remarkably low mean squared error (MSE) and a high coefficient of determination (*R*), providing strong evidence for the viability of this approach at an industrial scale. Our results represent a significant input in sustainable biodiesel production and underscore the enormous potential of natural heterogeneous catalysts derived from waste snail shells for achieving sustainable and environmentally friendly biodiesel production.

## Abstract 2

This study presents an approach to produce biodiesel from *Elaeis guineensis* using natural heterogeneous catalysts derived from raw, calcined, and acid-activated forms of waste snail shells.

## Graphical abstract

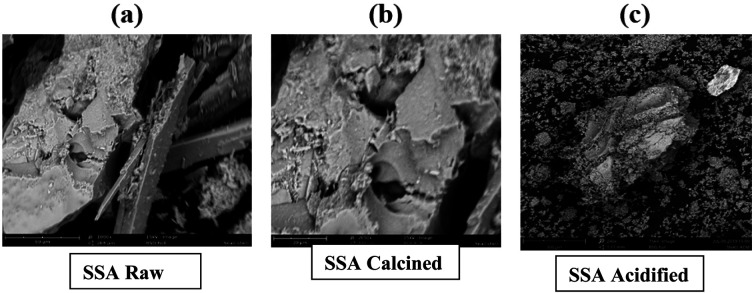

## 1 Introduction

Biodiesel production, exploration, and application in diesel engines have significantly impacted the discovery of sustainable and creative energy sources.^1^ Most research has focused on using indigenous sources to develop green and cleaner technologies that can mitigate the harm caused by fossil fuels and promote sustainable development.^2^ In recent years, alternative fuels for diesel substitution have been investigated to reduce reliance on petroleum-based energy and limit environmental pollution.^3^ The pollutants and emissions from fossil fuel usage have caused severe harm to the environment. Consequently, reducing greenhouse gas emissions to preserve the ecosystem was one of the key themes at the 2016 United Nations climate conference in Marrakesh.^4^ Therefore, we must not be complacent about the rising trend of environmental contamination.^5^ Scientists are increasingly searching for alternative fuels due to the depletion of fossil fuels, environmental concerns, and the continued rise in fossil fuel prices.

Biodiesel is considered a viable alternative to diesel in transportation, and demand for biodiesel is increasing. Biodiesel is oxygenated, non-toxic, less polluting, and biodegradable. In addition, its engine usage does not require any modifications.^6^ Worldwide biodiesel production in 2014 was approximately 25.2 million tons, with the European Union (EU) accounting for 37.1%, the United States for 16.7%, Brazil at 11.9%, and Argentina at 10.7%.^7^ Biodiesel has been established for use as a blend with diesel in transportation diesel engines. The United States diesel standard, ASTM D975, allows for up to 5% biodiesel without classifying it as a biodiesel blend.^8^ Biodiesel is known to have a high oxygen content, which is a critical quality for efficient burning. To further increase the oxygenation characteristic of biodiesel, several additives such as methanol, diethyl ether, ethanol, butanol, pentanol, diglyme, dimethyl ether (DME), and diethylene glycol methyl ether (DGM), among others, have been combined with the produced triglyceride to help reduce engine emissions. The clean-burning, low environmental pollution, and unique carbon advantages of palm biodiesel have attracted the interest of industrialists and increased public interest in developing sustainable green energy sources.^3,9^

The availability of feedstocks is a significant factor in determining the economic feasibility of biodiesel production, accounting for nearly 80% of the total cost of biodiesel. Numerous efforts have been made to identify low-cost feedstocks available throughout the year. The type and concentration of fatty acid in vegetable oils vary significantly, and the vegetable oil's composition depends on the choice of catalyst.^9,10^*Elaeis guineensis*, commonly known as the African oil palm, is an upright, one-stemmed palm tree that can grow up to 20–30 meters tall and has an adventitious root system.^11^ It has a total saturated fatty acid content of 87.92% and a total unsaturated fatty acid content of 12.08%.^12^ Vegetable oils have higher viscosity and lower volatility than diesel fuel.

This research focuses on the performance evaluation of *Elaeis guineensis* seed oils as biodiesel feedstock and the potential of deriving efficient natural solid catalysts for biodiesel production. This will complete the information gap of utilising other feedstocks to diversify feedstock in our local environment for their bio-fuel property and usability.

## 2 Materials and method

### 2.1 Materials

All reagents were purchased from Sigma Aldrich and used without further purification. The flashpoint of the biodiesel was measured using a Pensky Martens flashpoint tester, and the Soxhlet extractor was used to extract the oil from *Elaeis guineensis* seeds. Calcination was carried out using a Muffle furnace. The burning capacity of the biodiesel was evaluated using the TECHNO R175A diesel engine. The densities and viscosities of the seed oil and ester blends were measured using a digital density meter (model AP PAAR DMA 35) and a viscometer. Fourier-transform infrared spectroscopy (FTIR) was used to analyze the functional groups in the catalyst and seed oil. The catalyst morphology was examined using a spectrophotometer (8400SSHIMADZU) and Emission Scanning Electron Microscope (JSM-670IF). The components of the produced ester were identified using Gas chromatography-mass spectrometry (GC-MS) analyses using a QP 2010 SE instrument from Shimadzu, Japan. The separation was achieved on a Restek Rt × 5 ms column with a film thickness 0.32. The GC operating conditions were as follows: the temperature was held at 90 °C for 1 min, then increased from 90 °C to 150 °C at a rate of 13 min, followed by a final isothermal hold at 300 °C for 2 min. Helium was used as the carrier gas. The sample was injected in the split mode with the injector temperature of 250 °C. The mass spectrometer operated in the electron impact mode at 70 eV ionization energy, scanning from 45 to 700 Da. Data acquisition and processing were performed using ChemStation software.

### 2.2 Sample collection and oil extraction

Raw seeds of *Elaeis guineensis* were purchased from a local retailer in Ogbete main market, Enugu, Enugu State, Nigeria. The sample was sun-dried before being pulverized in an industrial blender. The resulting powder was sieved using a mesh size of 80 μm. The dried sample was then weighed into semi-permeable cotton material in the thimble of a Soxhlet extractor (500 mL). The Soxhlet extractor was connected to a condenser fixed to a round-bottom flask containing *n*-hexane (400 mL). The Soxhlet extraction system was refluxed at 70 °C until all the oil had been extracted from the sample (approximately 6 h). The solvent in the oil–solvent mixture was removed *in vacuo*, and the remaining solvent in the oil was distilled at 50 °C. The weight of the extracted oil was determined, and the percentage yield was calculated.

### 2.3 Catalyst preparation, characterisation and regeneration

The snail shell was first washed, dried in an oven for 2 h at 120 °C and ground into a fine powder. The resulting powder was then used to obtain the snail shell ash (SSA). Specifically, a sample of the dried SSA weighing 5 g was calcined at 800 °C for 3 h, while another sample of 10 g was acidified with 10 mL of concentrated sulphuric acid. The acidified SSA was then rinsed repeatedly with distilled water until the pH was 7. Finally, both samples were dried thoroughly in an oven at 120 °C and characterized using SEM and FTIR.

Regeneration of the catalyst was achieved by re-calcination and re-acidification after each run. The oil was removed using separating funnel and the bottom layer containing the catalyst was filtered under suction, dried completely and the regenerated catalyst weighed.

### 2.4 Biodiesel production (transesterification)

Transesterification reactions were conducted to produce the methyl and ethyl esters. Methanol/ethanol and oil were added in a ratio of 6 : 1 to 14 : 1, along with a modified catalyst (0.75–3.75 g) as specified for each experiment. The mixture was pre-stirred for 10 min to ensure proper dissolution of the catalyst. Subsequently, 50 mL of oil containing fatty acids was added and stirred at the required temperature for the specified time of 30–150 min at a specific temperature range of 30–70 °C.

Once the reaction was completed, the mixture was transferred to a separating funnel and allowed to stand for 12 h to allow for glycerol separation. The layers were then separated, and the resulting methyl and ethyl ester (biodiesel) were washed with hot water. The biodiesel was transferred into a beaker (250 mL) and heated to 105 °C to remove moisture. Finally, it was allowed to cool to room temperature.

### 2.5 Effect of process parameters

The influence of process parameters (quantity of catalyst, time, temperature, methanol/oil ratio, and agitation speed) was investigated using standard procedures.

### 2.6 Engine performance test

The biodiesel samples were analysed for engine fuel consumption rate, emission level, powering capabilities, and performance in comparison to a TECHNO R175A diesel engine. The consumption rate was determined by passing each sample (50 mL) at specific intervals. To test the emission level, the fumes produced by each sample during the engine performance test were passed through a conical flask containing 25 mL of 1 M sodium hydroxide solution. The resultant solution was titrated against a 1 M hydrochloric acid solution. The acidity of the vapor was calculated using the following formula:

1 Acidity of fumes = (*a* − *b*) × (molar mass of NaOH) × (molarity of NaOH)

The rate of fuel consumption was calculated as follows:2
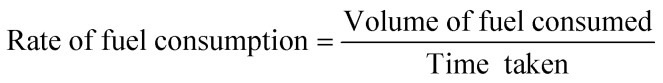


## 3 Results and discussion

### 3.1 Physicochemical properties

The physicochemical parameters of the extracted *Elaeis guineensis* seed oil (ESO) are presented in Table 1. The acidic compounds detected in the biodiesel might have originated from residual mineral acids from the production process, residual free fatty acids from the hydrolysis or post-hydrolysis process of the esters, or the oxidation of by-products in the form of other organic acids.^13^ The acid value (AV) of *E. guineensis* was found to be 0.710 mg_KOH_ g^−1^, which falls within the standard range of 0.5–0.8 (ASTM D6751 standard). Therefore, the oil was suitable for direct conversion into biodiesel using the base-catalysed transesterification procedure.^14^ Oils with high free fatty acid levels can deactivate the base catalyst, leading to soap formation and reduced biodiesel yield.^15^

**Table 1:** Physicochemical properties of the extracted oil

Properties	*E. guineensis*
Colour	Pale yellow
Yield (%)	58.87
Moisture (%)	5.50
Kinematic viscosity @ 40 °C (mm^2^ s^−1^)	14.96
Refractive index @ 29 °C	1.56
Energy value (kJ kg^−1^)	44 147
Acid value (mg_KOH_ g^−1^)	0.60
Saponification (mg_KOH_ kg^−1^)	248.00
Peroxide value (meq. kg^−1^)	1.21
Iodine value (g/100 g of oil)	52.00
Molecular weight (g mol^−1^)	840.54
Flash point (°C)	247.00
Cloud point (°C)	10.50
Pour point (°C)	5.00
Fire point (°C)	256.00
Density (g mL^−1^)	0.91

The iodine value of a fatty acid is a measure of its unsaturation, which in turn depends on the type and ester content of the feedstocks used in biodiesel production. A higher iodine value indicates a higher degree of unsaturation in the oil. Fatty acids with higher degrees of unsaturation are more susceptible to lipid peroxidation (rancidity), which can be prevented using antioxidants. Biodiesel with a high iodine content (115–120) can polymerise to produce deposits on injector nozzles, piston rings, and piston ring grooves. The higher degree of unsaturation in fatty acids enhances their tendency to polymerise.^16^ Therefore, biodiesel with high iodine content is less stable at higher temperatures. The iodine value of *E. guineensis* was determined to be 52.0 (g/100 g), below the standards of 120 and 115 for Europe and Germany, respectively, for EN 14214 and DIN 5160.^17^

The kinematic viscosity directly affects the fuel performance, atomisation quality, and combustion. The kinematic viscosity of a commercial biodiesel sample (methyl soyate) is 4.09 mm^2^ s^−1^, while that of petrol-diesel fuels is 1.54 mm^2^ s^−1^.^18^ The kinematic viscosities of the oils were higher than that of petrol diesel (2.5–3.2 mm^2^ s^−1^ at 40 °C), possibly due to the fatty acid composition and molecular weight of the oils.^19^

### 3.2 Snail shell ash (SSA) catalysts characterisation

The results of the catalyst characterisation of the SSA catalyst (Table 2) indicate that the catalyst could serve as a promising source for biodiesel production. The SSA catalyst in its raw form exhibited a surface area value of 655.000 m^2^ g^−1^, while the acidified form showed a value of 720.000 m^2^ g^−1^, and the calcined form exhibited a value of 832.00 m^2^ g^−1^. The calcined form demonstrated superior characteristics compared to the raw and acidified forms, particularly with regards to its larger surface area.

**Table 2:** Characterisation of SSA catalyst

Parameter	Raw SSA	Acid-activated SSA	Calcined SSA
Moisture (%)	0.04	0.06	0.03
Bulk density (g cm^−3^)	0.62	0.48	0.46
pH	6.23	4.62	5.87
Surface area (m^2^ g^−1^)	655	720	832
Carbon (%)	3.72	1.04	1.86
Organic matter (%)	11.84	3.18	5.32
Loss on ignition (%)	8.79	3.23	0.62
Particle density (g cm^−3)^	1.04	0.89	0.42
Total porosity (%)	42.04	48.86	25.54
Ash (%)	2.32	1.22	0.67

**Fig. 1:**
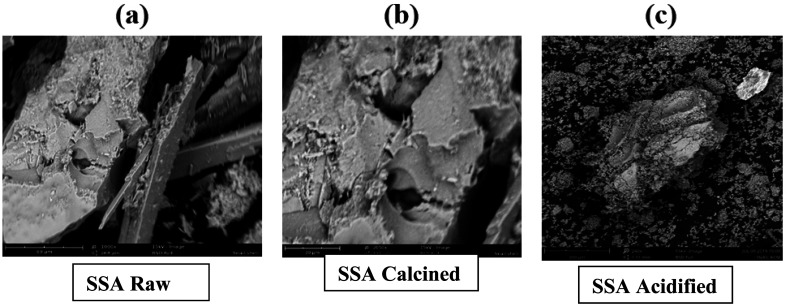
SEM images of SSA catalyst obtained from the raw, calcined, and acidified forms.

The SEM images of raw SSA showed the presence of a carbon structure covered with pores, while the calcined sample of SSA showed varying pore sizes and a broader surface area. The acidified sample displayed a picture of a collapsed carbon framework, resulting in the formation of irregular pores. Calcination provides additional space for the deposition of active sites, enhancing biodiesel yield by promoting the transesterification reaction (Fig. 1).^20^

### 3.3 Effect of process parameters on biofuel production from oil seed

Tables 3 and 4 present the effects of various process parameters, including catalyst concentration, time, temperature, methanol-to-oil ratio, and speed, on biodiesel production from *E. guineensis* (ESO) using methanol and ethanol for transesterification and snail shell ash (SSA) catalyst, respectively. The study found that the reaction rate for biofuel production increased with time, peaking at 120 and 150 min. Methanol yielded more than ethanol due to differences in activation energy, as predicted by Moser's reaction collision theory.^21^ The highest yield, 75.00%, was obtained from *E. guineensis* using calcined SSA *trans*-esterified with methanol at 150 min.

**Table 3:** Effect of process parameters on biodiesel yield using methanol for the transesterification process

Test parameters	Catalyst concentration (% wt. of catalyst)					Time (min)					Temperature (°C)					Methanol: oil ratio					Speed (rpm)				
	0.75	1.50	2.25	3.00	3.75	30	60	90	120	150	30	40	50	60	70	6 : 1	8 : 1	10 : 1	12 : 1	14 : 1	100	200	300	400	500
Biodiesel yield with raw SSA (vol%)	78	76	73	70	66	34	39	47	53	51	45	47	50	64	61	61	65	71	75	77	37	45	56	59	59
Biodiesel yield with acid-activated SSA (vol%)	80	76	72	70	68	42	48	62	65	65	46	51	59	62	73	63	71	76	75	78	45	62	71	76	75
Biodiesel yield with calcined SSA (vol%)	82	80	76	73	71	47	54	68	73	75	64	69	76	80	89	72	80	84	87	91	46	69	79	82	82

**Table 4:** Effect of process parameters on biodiesel yield using ethanol for the transesterification process

Test parameters	Catalyst concentration (% wt. of catalyst)					Time (min)					Temperature (°C)					Ethanol: oil ratio					Speed (rpm)				
	0.75	1.50	2.25	3.00	3.75	30	60	90	120	150	30	40	50	60	70	6 : 1	8 : 1	10 : 1	12 : 1	14 : 1	100	200	300	400	500
Biodiesel yield with raw SSA (vol%)	60	58	55	52	50	32	37	51	56	56	25	29	38	46	53	33	40	51	60	57	35	40	51	62	59
Biodiesel yield with acid-activated SSA (vol%)	68	65	62	62	61	38	48	60	62	61	31	36	46	52	57	40	57	67	72	69	41	58	70	74	74
Biodiesel yield with calcined SSA (vol%)	70	68	66	64	64	43	53	63	68	68	34	40	48	58	60	47	61	73	74	71	44	64	73	79	78

As depicted in Table 3, there is an inverse relationship between catalyst concentration and yield. Interestingly, a smaller quantity of the catalyst facilitates a superior yield. This outcome can be attributed to higher catalyst concentrations possessing greater activation energy, resulting in a slower reaction rate than their lower-concentration counterparts.

Temperature also impacted yield, with most samples achieving their highest yield at 60 °C and 70 °C. While the reaction could occur at ambient temperature, achieving a significant yield and conversion would take an extended period.^22^ The best yield, 89.00%, was obtained from *E. guineensis* using calcined SSA *trans*-esterified with methanol at 70 °C.

Methanol yielded more than ethanol due to its lower viscosity. The highest yield, 91.00%, was obtained from *E. guineensis* using calcined SSA *trans*-esterified with methanol at a 14 : 1 methanol-to-oil ratio.

Stirring speed also affected yield, with the best yield, 82.00%, obtained from *E. guineensis* using calcined SSA *trans*-esterified with methanol at 400 and 500 rpm.

In summary, using calcined SSA resulted in the highest average yield for seed oil due to the increased catalyst surface area and transformation of bulky molecules. Optimizing response parameters such as catalyst concentration, methanol-to-oil molar ratio, reaction temperature and duration, and agitation speed are recommended to improve production efficiency and reduce costs.

### 3.4 Kinetics of the reaction

Transesterification is a reversible process (eqn (1)) that can be influenced by process parameters, as studies have shown that their effects can increase the reaction rate.^23^ The rate is controlled only by the concentration of free fatty acids (FFAs).^14^ The reaction typically occurs in three steps, with pseudo-first-order kinetics observed when alcohol concentration is excessive.Step 1: Triglyceride + Alcohol ⇌ Diglyceride + EsterStep 2: Diglyceride + Alcohol ⇌ Monoglyceride + EsterStep 3: Monoglyceride + Alcohol ⇌ Glycerine + Ester

1 ROOH + R′OH ⇌ RCOOR′ + H_2_O

The rate can be expressed in terms of FFA disappearance (*A*) (eqn (2)), and eqn (3) can be used to describe the fluctuation in substrate concentration over time,^23^ where [*A*]_0_ and [*A*]_*t*_ are the initial and final concentrations of FFAs (mg_KOH_) after a time (*t*).

2 −*d*[*A*]d*t* = *k*[*A*]

Second-order kinetics can also be observed in transesterification reactions when the alcohol-to-FFA ratio is low (eqn (3)), and the rate is determined by the concentrations of both reactants (alcohol and FFAs) (eqn (4)).

3 ln[*A*]*t* = −*k*_*t*_ + ln[*A*]_0_

4 −*d*[*A*]d*t* = *k*_2_[*A*][ROH]

This study aims to verify the transesterification reaction sequence at an alcohol/oil ratio of 12 : 1, commonly used in commercial biodiesel production.^22^ The experimental data were fitted into zero, first, and second-order kinetic models and the best fit was selected. A plot was used to confirm the order of responses, and the slope equalled the rate constant (*k*). Table 5 shows the rate constant and coefficient of determination (*R*^2^) of methylation and ethylation of the base oil using an SSA catalyst. This research can contribute to the optimization of the transesterification process and improve commercial biodiesel synthesis.

**Table 5:** Overall rate constants and the coefficient of determination (*R*^2^) for methylation and ethylation of the base oil using SSA

Temp. (°C)	Methylation						Ethylation					
	Zero order	*R* ^2^	1st order, *K*_1_ (min^−1^)	*R* ^2^	2nd order, *K*_1_ (g mg_KOH_^−1^ min^−1^)	*R* ^2^	Zero order	*R* ^2^	1st order, *K*_1_ (min^−1^)	*R* ^2^	2nd order, *K*_1_ (g mg_KOH_^−1^ min^−1^)	*R* ^2^
30	0.004	0.833	0.002	0.869	0.005	0.871	0.002	0.908	0.003	0.921	0.027	0.935
50	0.006	0.957	0.012	0.942	0.021	0.925	0.005	0.964	0.009	0.921	0.011	0.952
70	0.004	1.000	0.006	0.993	0.009	0.992	0.005	0.964	0.007	0.953	0.011	0.956

The adequacy of the response to the related kinetic model was determined using the coefficient of determination (*R*^2^),^24^ with higher *R*^2^ values indicating higher confidence levels in the response's conformity. The reaction order is an essential factor in determining the relationship between concentration and rate and whether the reaction is accelerated or slowed down by the concentration.^24^

**Fig. 2:**
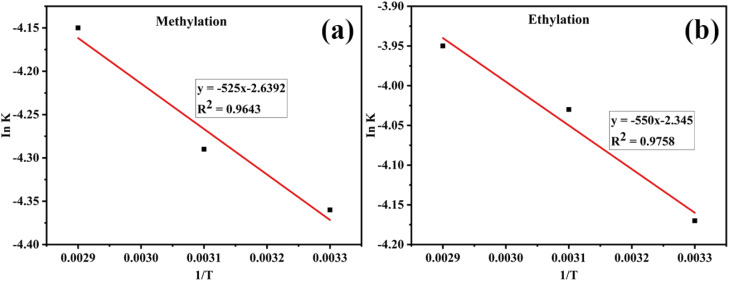
Determination of activation energy from the plot of ln *k versus* 1/*T* for (a) methylation and (b) ethylation.

The obtained constant rate values (Table 6) were used to determine the activation energy (*E*_a_) using the Arrhenius equation, represented by a plot of Ink *versus* the reciprocal of absolute temperature (Fig. 2).ln *k* = (−*E*_a_/*R*)/*T* + *A*

**Table 6:** Determination of activation energy for the second-order methyl and ethyl transesterification of *Elaeis guineensis* base oil

Temp. (K)	Methylation			Ethylation		
	1/*T* (K^−1^)	*K*	ln *K*	1/*T* (K^−1^)	*K*	ln *K*
303	0.0033	0.0128	4.36	0.0033	0.0155	−4.17
323	0.0031	0.0137	−4.29	0.0031	0.0177	−4.03
343	0.0029	0.0157	−4.15	0.0029	0.0193	−3.95

Activation energy is the minimum energy required by reacting molecules for a reaction. *E*_a_ represents the activation energy, *R* denotes the gas constant, *k* is the rate constant, and A is a constant. The activation energy for the methyl transesterification of the base oil sample using a snail shell catalyst was found to be 43.65 kJ mol^−1^. In comparison, ethylation transesterification was 45.73 kJ mol^−1^ for the snail shell catalyst.

#### 3.4.1 Determination of activation energy from the plot of ln *k versus* 1/*T* for (a) methylation

Slope = −*E*_a_/*R* −525 = *E*_a_/*R* *R* = 8.314 J K^−1^ mol^−1^ *E*_a_ = −(−525) (8.314 J K^−1^ mol^−1^) = 4365 J *E*_a_ = 43.65 kJ mol^−1^

#### 3.4.2 Determination of activation energy from the plot of ln *k versus* 1/*T* for ethylation

Slope = −*E*_a_/*R* −550 = *E*_a_/*R* *R* = 8.314 J K^−1^ mol^−1^ *E*_a_ = −(−550) (8.314 J K^−1^ mol^−1^) = 4573 *J* = 45.73 kJ mol^−1^

The results showed that the activation energy for methylation was 43.65 kJ mol^−1^, while ethylation was 45.73 kJ mol^−1^. The higher activation energy for ethylation, especially for the sample using a snail shell catalyst, suggests that it requires more energy for the reacting molecules to initiate the reaction than methylation.

### 3.5 Catalyst reusability

We investigated the reusability of the catalyst for subsequent transesterification runs under the same operating conditions, *i.e.*, a 14 : 1 methanol to oil ratio, 4 wt% catalyst loading, and 2 h reaction time. As shown in Table 7, our results revealed a significant decline in biodiesel yield, which was only evident after the fifth reaction cycle. During the first catalyst regeneration runs, the calcined SSA catalyst yielded the highest conversion yield (89.65%) and retained the most significant amount of catalyst (2.25 g). However, with increasing regeneration runs, the yield decreased, and the calcined catalyst showed a higher percentage conversion per run than the acid-activated catalyst during the same run. This observation may be attributed to the deposition of unreacted oil and glycerol components on the catalyst's active sites, leading to the coverage of the sites and hindering the catalyst's effectiveness.^10^

**Table 7:** Regeneration of SSA catalyst

Regeneration runs	Amount of catalyst remaining after each use (g)	Conversion yield of *E. guineensis* for the acid-activated sample (vol%)	Conversion yield of *E. guineensis* for the calcined sample (vol%)
1	2.25	80.23	89.65
2	1.78	79.78	87.26
3	1.20	77.9	81.58
4	0.75	76.25	75.24
5	0.25	65.98	72.75

### 3.6 Fuel properties of *E. guineensis* seed oils and their derivatives

The results of the fuel samples indicate that most of them fell within the standard diesel ASTM range. Among the samples, Sample 2 exhibited the best fuel properties in terms of viscosity, which was produced by *trans*-esterifying *E. guineensis* with methanol using an acid activated SSA catalyst. The viscosity of Sample 2 decreased to 0.30 mm^2^ s^−1^ with continuous blending at higher temperature (100 °C), suggesting that blending petrol diesel with biodiesel can enhance the viscosity, thereby ensuring their compatibility with modern diesel engines equipped with fuel injection systems that are sensitive to changes in viscosity.^25^ Moreover, the samples obtained from methyl ester showed better fuel properties than those obtained from ethyl ester.

**Table 8:** Fuel properties of *E. guineensis* seed oil and its derivatives using the SSA catalyst^a^^,^^b^

Sample		Density at 28 °C	Flashpoint (°C)	Calorific value (J kg^−1^)	Pour point (°C)	Viscosity at 40 °C (mm^2^ s^−1^)	Viscosity at 70 °C (mm^2^ s^−1^)	Viscosity at 100 °C (mm^2^ s^−1^)	Acid value (mg_KOH_ g^−1^)
Sample 1/SSA/calcined/methyl ester	B_100_	0.896	174	44 357	6.0	7.5	3.8	3.6	0.7
	B_80_	0.886	152	44 938	5.5	6.8	2.5	3.2	0.6
	B_60_	0.874	138	44 938	4.5	5.5	1.7	2.2	0.5
	B_40_	0.85	127	45 334	3.5	4.75	1.2	0.9	0.4
	B_20_	0.845	114	45 334	2.0	3.2	0.7	0.4	0.3
Sample 2/SSA/acid-activated/methyl ester	B_100_	0.891	169	44 543	5.0	7.0	3.2	3.3	1.6
	B_80_	0.888	148	44 543	4.5	6.2	2.7	2.4	1.4
	B_60_	0.883	133	44 752	3.5	5.8	1.9	1.8	1.2
	B_40_	0.866	121	44 938	3.0	4.5	1.0	1.0	0.8
	B_20_	0.844	112	45 520	2.0	3.0	0.5	0.3	0.6
Sample 3/SSA/calcined/ethyl ester	B_100_	0.895	178	44 357	5.0	7.3	3.5	3.8	0.8
	B_80_	0.878	159	44 752	4.0	6.5	2.8	3.1	0.7
	B_60_	0.865	143	44 938	3.0	5.2	2.0	2.3	0.6
	B_40_	0.856	130	45 124	2.5	4.5	1.5	1.5	0.5
	B_20_	0.833	116	45 729	1.5	3.8	0.9	0.8	0.4
Sample 4/SSA/acid-activated/ethyl ester	B_100_	0.892	172	44 543	5.5	6.8	3.1	3.0	1.5
	B_80_	0.877	150	44 752	5.0	6.2	2.7	2.8	1.3
	B_60_	0.872	136	44 938	4.0	5.8	1.8	2.2	1.0
	B_40_	0.853	124	45 334	3.0	4.2	1.3	1.5	0.9
	B_20_	0.842	110	45 520	2.0	3.5	0.8	0.7	0.7
ASTM limit		0.8–0.9	100 (min.)	NA	16 (max.)	1.9–6.0	NA	NA	0.80 (max.)

a NA = not available; min. = minimum; max. = maximum.

b B20–B100 = blend of 20% biodiesel and 80% traditional diesel and so on.

### 3.7 Metal composition of the seed oils and their derivative

The heavy metal composition analysis of the seed oils, methyl esters, and ethyl esters of the feedstocks revealed that all the samples contained trace amounts of heavy metals (Table 9). The levels of heavy metals were lower than those found in petroleum diesel, suggesting that none of the fuel samples would pose a corrosion risk to the injector and piston chambers of diesel engines.^26^

**Table 9:** Metal composition of the fuel samples^a^

Metals	Diesel	*E. guineensis* oil	EME	EEE
Pb	0.45	0.32	0.22	0.22
Cd	1.03	0.39	0.23	0.23
Hg	0.79	0.57	0.13	0.57
As	1.89	1.1	0.61	0.71
Bi	2.06	0.33	0.23	0.22
Ag	1.84	1.13	1.8	0.89
Sn	0.79	0.52	0.23	0.33
Cu	0.3	1.7	1.02	0.6
Zn	1.63	0.79	0.22	0.22
Ni	3.52	0.76	0.12	0.17

a EME = methyl esters of *E. guineensis*; EEE = ethyl esters of *E. guineensis*.

### 3.8 Acidity concentration of fumes from combustion of the methyl ester and diesel samples

We investigated the combustion properties and emissions of biodiesel and diesel samples in a compression ignition direct injection engine. The combustion test results showed that all the samples (Table 10) could simultaneously power the Techno R175A diesel engine. During combustion, a small portion of the fuel premixes with the air before the bulk combustion event, which occurs through a mixing-controlled process of flame envelopment of the fuel spray.

**Table 10:** Concentration of acidic fumes of different samples (methyl esters) for engine performance tests^a^

Sample	Concentration (g dm^−3^)
Diesel	0.0045
B_100_	0.0012
B_80_	0.0015
B_60_	0.0014
B_40_	0.0012
B_20_	0.0022

a B20–B100 = blend of 20% biodiesel and 80% traditional diesel, and so on.

The samples emitted higher emissions with a less irritating smell and darker emissions than diesel. However, using biofuels as an alternative to fossil fuels could reduce air quality degradation caused by combustion. The results also confirmed that the acid concentrations in the exhaust engine were lower than those of hydrocarbon diesel. However, as the proportion of diesel blended into the biodiesel increased, the acid concentrations in the exhaust smoke increased.

The results of this study provide valuable insights into the potential use of biodiesel as an alternative fuel and highlight the importance of reducing emissions from fossil fuel combustion. B100 (Table 10) demonstrated the most significant reduction in emissions, possibly due to the biodiesel's oxygen content, which facilitated complete combustion and decreased hydrocarbon and monoxide emissions. The findings suggest that engine performance could be improved by further diluting diesel with *trans*-esterified vegetable oils.^19,27,28^

### 3.9 Modelling and optimisation by artificial neural network

The performance of differently prepared samples of SSA for biodiesel yield in the transesterification process of *E. guineensis* seed oil was evaluated using artificial neural network (ANN) modelling and optimization. The ANN was also used to develop a system that relates process variables to biodiesel yield.

### 3.10 Experimental design

The experimental design involved varying the catalyst concentrations, agitation speed, methanol/oil molar ratio, and temperature and recording the resulting fatty acid methyl ester (FAME) yield. A total of twenty-five samples were analysed, and the data sets were input into the ANN toolkit for kinetic modelling of the process. The dependent variable in this study was the percent FAME yield, while the independent variables were catalyst concentration, methanol/oil molar ratio, agitation speed, and temperature.

Table 11 presents the FAME yield for the varied catalyst concentrations, agitation speed, molar ratio, and temperature. The results of this study demonstrate that the ANN model successfully predicted the FAME yield for the different process variables. The use of SSA has been shown to be an efficient and effective method for producing biodiesel. This study highlights the potential of ANN modelling to optimise the biodiesel production process band and improve its overall efficiency.

**Table 11:** FAME yield from *E. guineensis* oil using the three forms of SSA^a^

Std run	A : A	Input variable	Experimental results		
		Catalyst conc. (% wt)	Yield 1/raw (vol%)	Yield 2/acid-activated (vol%)	Yield 3/calcined (vol%)
1	9	0.75	55	72	73
2	13	3.75	55	72	73
3	15	0.75	65	72	73
4	23	3.75	65	72	73
5	5	0.75	55	78	73
6	1	3.75	55	78	73
7	12	0.75	65	78	73
8	21	3.75	65	78	73
9	2	0.75	55	72	82
10	4	3.75	55	72	82
11	7	0.75	65	72	82
12	17	3.75	65	72	82
13	19	0.75	55	78	82
14	18	3.75	55	78	82
15	10	0.75	65	78	82
16	22	3.75	65	78	82
17	24	−0.75	60	75	77.5
18	8	5.25	60	75	77.5
19	6	2.25	50	75	77.5
20	20	2.25	70	75	77.5
21	14	2.25	60	69	77.5
22	25	2.25	60	81	77.5
23	11	2.25	60	75	68.5
24	3	2.25	60	75	86.5
25	16	2.25	60	75	77.5

a Temperature = 55 °C; mole ratio = 10 : 1; time = 3 h; speed = 300 rpm.

### 3.11 ANN model validation

**Fig. 3:**
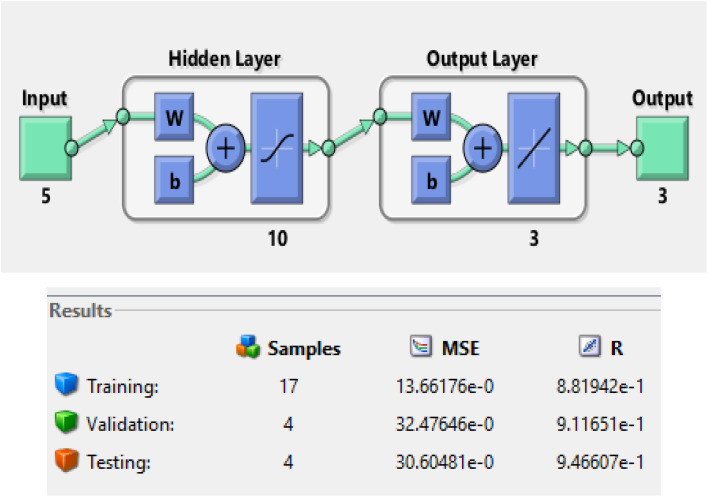
Architecture of the ANN network.

A model of an Artificial Neural Network (ANN) was developed and validated to predict biodiesel yield using SSA catalysts. The model was trained using experimental data obtained from Table 8 and implemented with the Levenberg–Marquardt (LM) ANN fitting tool and a Logistic Sigmoid Activation Transfer Function 5-10-3 model. The architecture of the ANN Network for training, validation, and testing is shown in Fig. 3.

**Fig. 4:**
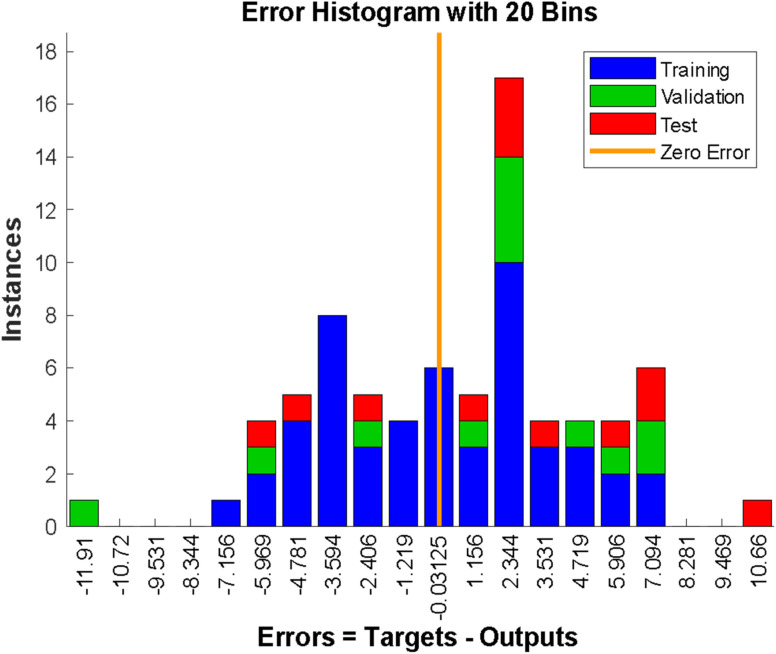
Error histogram of the model.

Fig. 4 illustrates the basic building block of an ANN, which is an artificial neuron. The inputs are weighted at the entrance of the artificial neuron, and the sum function in the middle section sums all weighted inputs and bias. At the exit of the artificial neuron, the sum of previously weighted inputs and bias passes through the activation function to generate a response. The mean square error value obtained was significant, and the *R* values obtained for training, testing, and validation were 0.875, 0.941, and 0.929, respectively. The overall value achieved was 0.882, indicating a good agreement between the anticipated ANN solution model and the result acquired from experimental biodiesel yield.

**Fig. 5:**
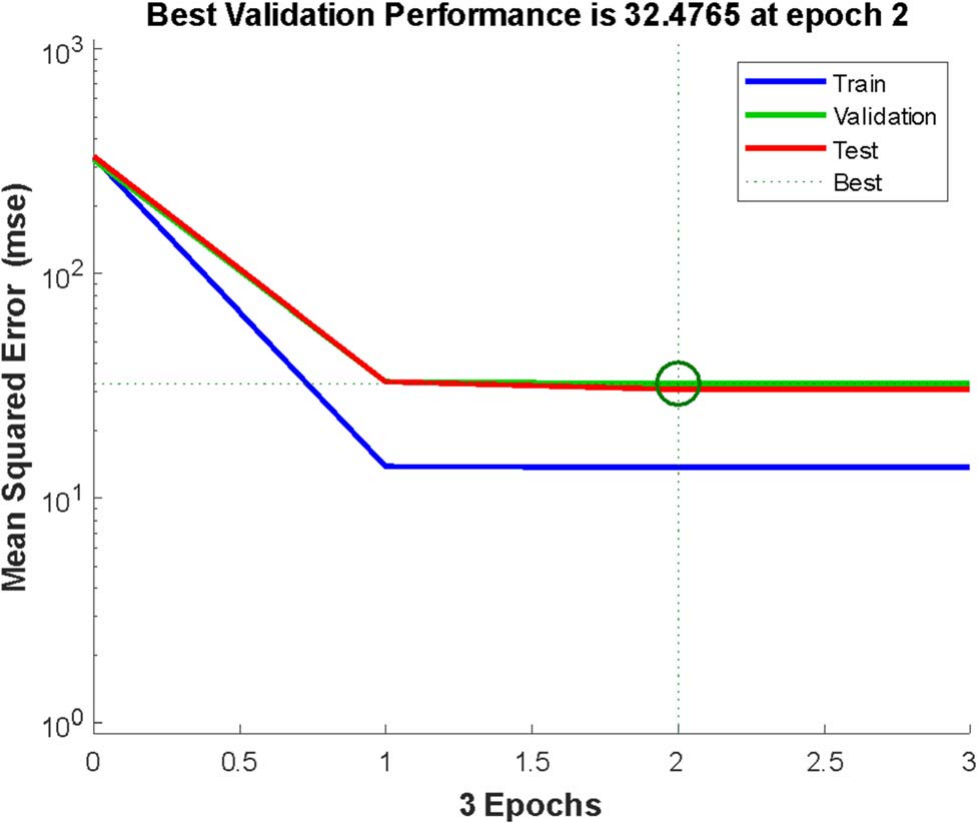
Validation performance.

The error histogram presented in Fig. 5 shows the training, validation, testing, and negligible error. The back-propagation algorithm was used in this context, and the slightest error of −0.003125 was observed. Fig. 5 shows the best validation performance at epoch 2, obtained by training the neural network with all the training data for one cycle, using all the data once for the forward and backward pass to get the best validation performance (Fig. 5).

**Fig. 6:**
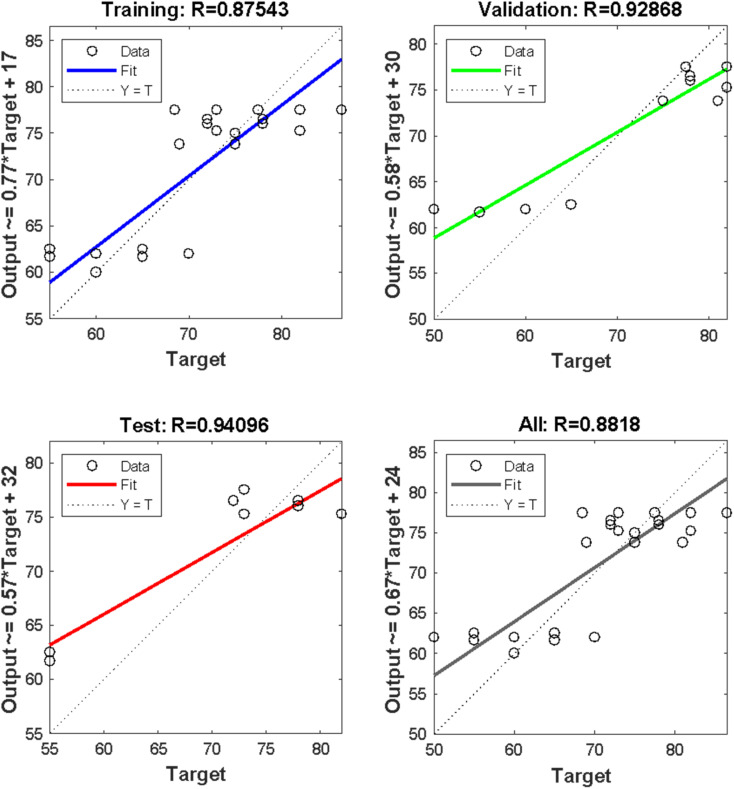
ANN regression plot for training, testing and validation with their various *R*-values.

Regression plots for training, validation, test, and overall plot are presented in Fig. 6, using the result generated from the experiment for SSA raw, calcined, and acidified catalyst. The predicted values in Table 12 were similar to the experimental results obtained from Table 11, demonstrating the efficacy of using the ANN predictive tool to simulate the production of *E. guineensis* using an SSA catalyst in 3 different forms (raw, calcined, and acid-activated).

**Table 12:** ANN solution for predicted values *E. guineensis* for the three forms of SSA (raw, acid-activated, and calcined)

Std run	A : A	Input variable	Output value			ANN predicted value		
		Catalyst conc. (% wt. of oil)	Yield 1/raw (vol%)	Yield 2/acid-activated (vol%)	Yield 3/calcined (vol%)	Yield 1/raw (vol%)	Yield 2/acid-activated (vol%)	Yield 3/calcined (vol%)
1	9	0.75	60.03	75.94	76.04	60.03	75.94	76.04
2	13	3.75	61.01	75.61	74.82	61.01	75.61	74.82
3	15	0.75	60.03	75.94	76.04	60.03	75.94	76.04
4	23	3.75	61.01	75.61	74.82	61.01	75.61	74.82
5	5	0.75	60.03	75.94	76.04	60.03	75.94	76.04
6	1	3.75	61.01	75.61	74.82	61.01	75.61	74.82
7	12	0.75	60.03	75.94	76.04	60.03	75.94	76.04
8	21	3.75	61.01	75.61	74.82	61.01	75.61	74.82
9	2	0.75	60.03	75.94	76.04	60.03	75.94	76.04
10	4	3.75	61.01	75.61	74.82	61.01	75.61	74.82
11	7	0.75	60.03	75.94	76.04	60.03	75.94	76.04
12	17	3.75	61.01	75.61	74.82	61.01	75.61	74.82
13	19	0.75	60.03	75.94	76.04	60.03	75.94	76.04
14	18	3.75	61.01	75.61	74.82	61.01	75.61	74.82
15	10	0.75	60.03	75.94	76.04	60.03	75.94	76.04
16	22	3.75	61.01	75.61	74.82	61.01	75.61	74.82
17	24	−0.75	60.02	75.02	77.51	60.02	75.02	77.51
18	8	5.25	60.01	75.14	77.51	60.01	75.14	77.51
19	6	2.25	62.50	73.50	75.25	62.50	73.50	75.25
20	20	2.25	62.50	73.50	75.25	62.50	73.50	75.25
21	14	2.25	62.50	73.50	75.25	62.50	73.50	75.25
22	25	2.25	62.50	73.50	75.25	62.50	73.50	75.25
23	11	2.25	62.50	73.50	75.25	62.50	73.50	75.25
24	3	2.25	62.50	73.50	75.25	62.50	73.50	75.25
25	16	2.25	62.50	73.50	75.25	62.50	73.50	75.25

## 4 Conclusion

This study demonstrates the potential of *Elaeis guineensis* as a cost-effective and widely available candidate for biodiesel production. With an oil yield of 56.87%, this crop can be used for commercial production of biodiesel. The catalyst used in the transesterification reaction exhibited varied activity based on their pore sizes, with the calcined form showing a larger surface area. The study also found that the biodiesel produced from *Elaeis guineensis* and its blends met the ASTM D6751 limits for fuel properties, making it suitable for biodiesel production. Furthermore, the heavy metal levels were within acceptable limits. The statistical tools used in the analysis showed a high correlation between the experimental and predicted values, indicating the accuracy of the model. These findings support large-scale biodiesel production, which could contribute significantly to the economy. Additionally, the study suggests the potential for utilizing the process by-product glycerol in various industrial applications. Overall, these results provide valuable insights into the development of alternative fuels and have important implications for sustainable energy production.

## List of abbreviations

SSA: Snail shell ash
FFA: Free fatty acid
FAME: Fatty acid methyl ester
ANN: Artificial neural network
MATLAB: Mathematics laboratory
MSE: Mean square error

## Conflicts of interest

There are no conflicts to declare.

## Supplementary Material
